# Is Intraoperative Biopsy Necessary for Gastric Ulcer Perforation? A Systematic Review

**DOI:** 10.3390/jcm15145460

**Published:** 2026-07-13

**Authors:** Adem Tuncer, Cuneyt Kayaalp, Servet Karagul

**Affiliations:** 1 Department of General Surgery, Florence Nightingale Hospital, Demiroğlu Bilim University, Istanbul 34365, Turkey; 2 Department of General Surgery, Faculty of Medicine, Istanbul Atlas University, Istanbul 34403, Turkey

**Keywords:** gastric cancer, peptic ulcer perforation, stomach ulcer, stomach cancer

## Abstract

**Introduction:** Gastric ulcer perforation is a life-threatening surgical emergency in which delay is poorly tolerated. In this setting a specimen is taken from ulcer margin to detect possible malignancy risk. The purpose of the present systematic review is to evaluate the need of intraoperative biopsy in gastric ulcer perforations using current biopsy-proven malignancy rates and its effect on complications. **Methods:** The review was carried out in line with the PRISMA guidelines and was registered (INPLASY202650135). Reports describing adults who operated on for a perforated gastric ulcer were considered. The principal endpoint was detection of malignancy. Malignancy proportions were combined within a random-effects model after Freeman–Tukey double-arcsine transformation, and statistical heterogeneity was quantified with the I^2^ statistic. **Results:** Twelve reports were eligible for the review, amounting to 1122 patients. Of these, 953 individuals (64% men; average age 52 years) underwent sampling of the ulcer at operation. Twenty-seven patients had biopsy-proven malignancy on intraoperative histology; one additional patient had a false-negative intraoperative biopsy that was confirmed as malignant on postoperative endoscopy, so the biopsy-proven outcome was based on 27 events. The random-effects pooled proportion of biopsy-proven malignancy among patients with no previous gastric cancer diagnosis was 3.1% (95% CI 1.4–5.7%). There was substantial statistical heterogeneity between studies (I^2^ = 72%; Cochran Q = 39.8, df = 11, *p* < 0.001). Sampling was omitted in 169 patients, and complications in patients with and without biopsy were contrasted in a single report only. In that single retrospective study, both total complications (46.4% vs. 11.8%, *p* = 0.007) and Clavien–Dindo grade ≥ III complications (34.5% vs. 5.9%, *p* = 0.017) occurred more often after biopsy since this signal derives from one single-centre comparison and may be confounded by ulcer complexity, so it ought to be treated as a hypothesis to be tested rather than as an established effect. **Conclusions:** The available evidence does not support the routine biopsy of all perforated gastric ulcers during surgery. However, as the evidence base is almost entirely retrospective and the included studies did not categorize lesions by size or appearance, a selective biopsy or intraoperative frozen section strategy may still be justified for large, chronic, mass-like, or otherwise suspicious ulcers, particularly in elderly patients.

## 1. Introduction

Perforation of stomach is an emergency that requires immediate operative treatment to prevent sepsis, peritonitis, and death. The etiology of gastric perforation varies, including peptic ulcer disease, malignancy, trauma, iatrogenic injury, and rare conditions such as infections or vasculitis [1,2,3]. Although benign disease accounts for the majority of the cases, malignancy remains a critical concern, particularly in regions with a high prevalence of gastric cancer [4].

Whether tissue should be sampled while the perforation is being repaired remains contested. Proponents argue that biopsy aids in detecting malignancy early, guiding further oncologic management, while opponents suggest that it may unnecessarily prolong surgery without significantly altering immediate outcomes. Because the consequences for postoperative treatment and for prognosis are considerable, the question of whether routine sampling is required deserves a clear answer. Major society guidance reflects this uncertainty. The World Society of Emergency Surgery (WSES) guidelines on perforated peptic ulcer do not mandate routine biopsy of every perforation but recommend resection with intraoperative frozen-section examination for large gastric ulcers that raise suspicion of malignancy, when the patient’s condition allows [5]. Endoscopy society guidance (e.g., ESGE) similarly emphasizes histological confirmation of gastric ulcers in the elective setting and structured endoscopic follow-up to exclude malignancy, a principle that is difficult to apply during emergency perforation surgery. Framing our question against these recommendations highlights why the role of routine intraoperative biopsy in the emergency setting remains unsettled.

The present review was designed to evaluate the clinical utility, diagnostic yield, and management impact of intraoperative biopsy in perforated gastric ulcer to determine gastric cancer. By drawing the available data together, we aim to indicate evidence-based recommendations on whether routine biopsy should be standard practice or not.

## 2. Materials and Methods

### 2.1. Protocol and Registration

Reporting followed the Preferred Reporting Items for Systematic Reviews and Meta-Analyses (PRISMA) statement so that the process would be methodologically rigorous and transparent (Supplementary Materials: Table S1). A protocol was deposited with the International Platform of Registered Systematic Review and Meta-analysis Protocols (INPLASY; registration number INPLASY202650135) on 25 May 2026. No restriction was placed on the year, the country or the language of publication. Eligibility was framed according to PICOS, as set out below.

- Population: adults aged 18 years or older operated on for perforation of a gastric ulcer.
- Intervention: sampling of the ulcer at operation, whether by frozen section or by permanent histopathology.
- Comparator: operations in which no such sample was taken.
- Outcomes: the proportion of specimens revealing malignancy or other clinically important disease, and complications after surgery.
- Study Types: randomized controlled trials (RCTs), cohort and case–control designs, and case series containing at least ten patients.

We set aside studies of children, perforations of duodenal ulcers, single-case descriptions, editorials, and conference abstracts for which no full data existed. Reports written in languages other than English for which no translation could be obtained were also excluded, as were those in which the results of sampling or the outcomes were not clearly documented.

### 2.2. Search Strategies

**Figure 1:**
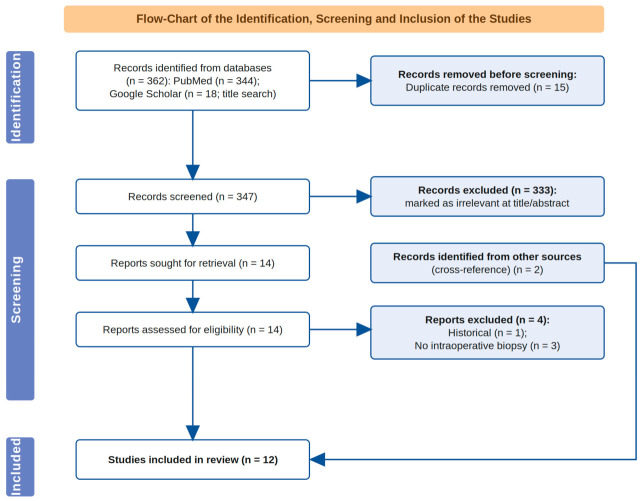
Preferred Reporting Items for Systematic Reviews and Meta-Analysis (PRISMA) flowchart.

PubMed/MEDLINE and Google Scholar were interrogated between 1 April and 1 May 2025, without limits on year, country, or language. The full PubMed search string was (gastric[Title]) AND (biopsy[Title]) AND (perforation[Title] OR perforations[Title] OR perforated[Title]); in Google Scholar, the equivalent title-restricted terms (allintitle: gastric biopsy perforation) were used. Bibliographies of the retrieved papers were then examined by hand for further eligible reports. The search was deliberately title-anchored to maximize specificity for the narrow clinical question; the resulting restriction to two databases and to title terms is acknowledged as a limitation in Section 4. Titles and abstracts were screened by two reviewers working independently, after which the retained papers were read in full (Figure 1). Disagreement was settled by discussion. Selection of studies, extraction of data and appraisal of bias were each undertaken in duplicate by the two reviewers, with disagreements resolved by discussion and, where necessary, adjudication by the senior author. The protocol was registered with INPLASY (INPLASY202650135). Since this study involved analyzing previously published data, no additional ethical approval was required.

From every included report we abstracted the characteristics of the study (first author, year, country, design and number of patients), the age and sex of the patients, the nature of the perforation (benign or malignant) and the complications recorded after operation. For quality and risk of bias, the Cochrane tool had been specified in advance for randomized trials and the Joanna Briggs Institute checklist for pure case series; since no randomized trial was found and every included report was observational, each study was instead appraised with the Newcastle–Ottawa Scale (NOS)—the cohort version for retrospective and prospective cohorts and the adaptation of Modesti et al. for cross-sectional designs. To avoid the inconsistent categories used previously, the total NOS score was graded uniformly as high quality/low risk (7–9), moderate quality/moderate risk (4–6), and low quality/high risk (0–3). Because the Modesti cross-sectional adaptation is scored out of ten rather than nine, the single cross-sectional study (Amoli) was rescaled to the nine-point NOS framework before applying the common grading thresholds, so that all studies are reported on a comparable 0–9 scale in Table 1.

### 2.3. Statistics

Study and patient characteristics were described with summary statistics. Inconsistency between the reports was expressed by the I^2^ statistic, values above 50% being taken to indicate substantial heterogeneity. The primary outcome (proportion of biopsy-proven malignancy) was pooled as a single-arm proportion. Because several studies reported zero events, raw proportions were stabilized with the Freeman–Tukey double-arcsine transformation before pooling, which allows zero-event studies to be retained without an arbitrary continuity correction; the pooled estimate was then back-transformed to a proportion with its 95% confidence interval. A DerSimonian–Laird random-effects model was used as the primary analysis given the anticipated clinical and methodological heterogeneity, with the fixed-effect estimate reported for comparison. Between-study heterogeneity was quantified by I^2^ and the Cochran Q test, and small-study effects were explored visually with a funnel plot. Study and patient characteristics were summarized with descriptive statistics; categorical variables were expressed as counts and proportions and continuous variables as means with standard deviations. Study-level data were entered into a spreadsheet (Excel 2021, Microsoft Corporation, Redmond, WA, USA), independently cross-checked by both reviewers, and exported for pooling. To explore the substantial heterogeneity, we performed pre-specified leave-one-out analyses and subgroup analyses by publication era (before 2000 vs. 2000 onward) and by geographic region. As a sensitivity analysis, the pooled proportion was recomputed after excluding non-peer-reviewed sources (one preprint and one postgraduate thesis). Given twelve studies, several zero-event studies, and a proportion outcome, formal tests for funnel-plot asymmetry were not performed and the plot was interpreted with caution. Pooling was performed in MedCalc (MedCalc^®^ Statistical Software version 20.118; MedCalc Software Ltd., Ostend, Belgium; https://www.medcalc.org; 2022). Where a report gave the median and range, the mean and standard deviation were estimated by the method of Hozo et al. [18].

## 3. Results

The two databases yielded 362 records. Once unsuitable papers had been removed, twelve reports comprising 1122 patients remained (Figure 1) [6,7,8,9,10,11,12,13,14,15,16,17]. Three originated in South Africa, two in the United States and two in India; the remainder came from Europe and from Asia. No randomized controlled trial was identified and the majority of the reports consisted of single-arm retrospective data. Their appraisal on the Newcastle–Ottawa Scale is presented in Table 1.

Under the uniform grading, two reports carried a low risk of bias (7/9) and the remaining ten a moderate risk (5–6/9); none fell into the high-risk band (≤3/9). Sampling of the ulcer at operation was performed in 953 patients (64% male, average age 52 years). Cancer was demonstrated in the intraoperative specimens of 27 patients, while in one further patient [10] the specimen was falsely negative and the diagnosis was established subsequently on endoscopic biopsy. Counting this delayed diagnosis, 28 patients in all proved to have malignant disease (Table 2). The primary outcome was defined as biopsy-proven malignancy detected on intraoperative histology and was therefore based on 27 events. The single postoperatively diagnosed case (Leeman [10]), which was a false negative on intraoperative biopsy, is excluded from this numerator; accordingly, Leeman is entered in Table 2 and the forest plot as 3 of 33 (9.1%) rather than 4 of 33 (12.1%). The 28-patient figure represents total malignancy (intraoperative plus postoperatively confirmed) and is reported separately to avoid conflating the two outcomes.

**Figure 2:**
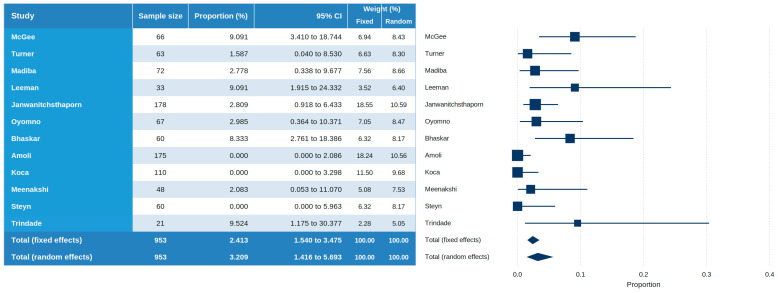
Proportion of cancer incidence of the studies.

**Figure 3:**
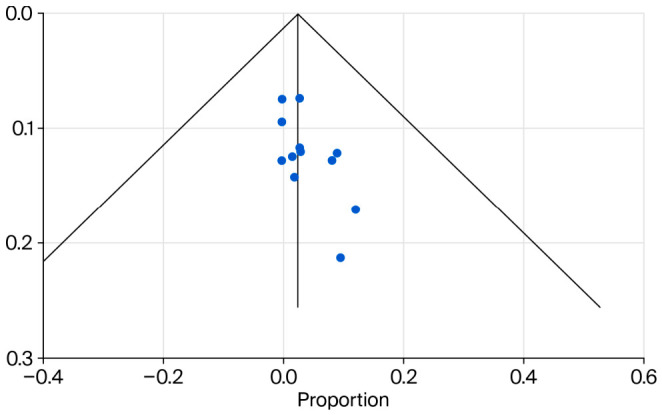
Funnel plot.

The random-effects pooled proportion of biopsy-proven malignancy among patients with no previous gastric cancer diagnosis was 3.1% (95% CI 1.4–5.7%), based on the corrected 27-event biopsy-proven numerator. There was substantial statistical heterogeneity between studies (I^2^ = 72%; Cochran Q = 39.8, df = 11, *p* < 0.001), as displayed in the forest plot (Figure 2). The funnel plot (Figure 3) appeared asymmetrical; however, with only twelve studies, several zero-event studies, and a proportion outcome, formal small-study-effect testing is unreliable, so this asymmetry should be interpreted cautiously and may largely reflect the wide between-study variation in reported malignancy rates (0% to 9.5%). Pre-specified analyses confirmed the robustness of the pooled estimate: a leave-one-out analysis kept the pooled proportion within a narrow range (approximately 2–3%) with no single study driving the result, and a sensitivity analysis excluding the non-peer-reviewed preprint and thesis left the estimate in the same low range and did not alter the conclusions. Subgroup analysis suggested a higher pooled proportion in the two pre-2000 studies than in the studies from 2000 onward, and variation across geographic regions, consistent with differences in era and background gastric-cancer epidemiology as drivers of the heterogeneity.

Sampling was not carried out in 169 patients, and only one report [9] set complications in sampled and unsampled patients side by side. In that study, both total complications (46.4% vs. 11.8%, *p* = 0.007) and Clavien–Dindo grade ≥ III complications (34.5% vs. 5.9%, *p* = 0.017) were more frequent in patients who underwent biopsy [9]. The no-biopsy group was considerably smaller than the biopsy group (169 vs. 953 patients overall, and the two arms within this single study were also unequal), and the comparison may be confounded by indication; these figures should therefore be read with caution.

## 4. Discussion

Sampling the ulcer at operation has long been customary in gastric perforation, the practice resting chiefly on the fear that a cancer will otherwise be overlooked. Data accumulating in recent years, however, indicate that malignancy complicates perforated gastric ulcer far less often than earlier reports implied, and that routine sampling is correspondingly difficult to defend. In the present review the pooled proportion of biopsy-proven cancer was approximately 3.1% among patients whose ulcers were sampled. This low incidence, taken together with the limited and uncertain morbidity signal associated with biopsy, suggests that routine intraoperative histopathological assessment of every perforated gastric ulcer may not be necessary; however, given the retrospective and heterogeneous evidence base, this should be read as support for a selective, individualized strategy rather than a blanket recommendation against biopsy.

Older series reported malignancy in as many as 9–23% of cases [19,20,21], and our figure stands in contrast to them. Several explanations may be offered for the high rates recorded in the past. The spread of endoscopy, and with it earlier diagnosis, is one. Another is that those retrospective studies drew on hospital records covering all gastric perforations, some of the patients already carrying a diagnosis of gastric cancer. None of the historical series based its figures on tissue taken during the operation.

Apart from the meagre diagnostic return, there is emerging evidence that excising tissue during emergency surgery for gastric perforation may add to postoperative morbidity. In a retrospective series of 135 patients, Koca et al. [9] recorded significantly more postoperative complications (46.4% vs. 11.8%, *p* = 0.007) and more severe ones (Clavien–Dindo ≥III: 34.5% vs. 5.9%, *p* = 0.017) among patients whose ulcers had been excised. Their proposed mechanism was that removal of tissue widens the defect, so that the sutures are placed under greater tension and leakage becomes more likely. Suture dehiscence did not differ significantly in their data, yet the trend towards more frequent reoperation (*p* = 0.089) points to a risk of clinical consequence. This evidence, however, comes from a single retrospective single-centre study, and the type, severity, and mechanism of the excess complications are not fully characterized. The morbidity signal would be more convincing if the complications were directly attributable to enlargement of the perforation, leakage, bleeding, or reoperation; if they instead reflected baseline patient severity, ulcer size, peritonitis, or selection bias, then biopsy may simply be a marker of more complex cases rather than an independent cause of harm. Confounding by indication is plausible, because surgeons may preferentially biopsy larger, more suspicious, or more complex ulcers, which independently increase complication risk. A more accurate summary is therefore that the available comparative evidence is limited to one retrospective study and suggests an association between biopsy and increased complications; this association may be influenced by selection bias and ulcer complexity and should not be taken to establish that biopsy itself increases morbidity. Accordingly, in the minority of benign-appearing perforations the limited and uncertain harm signal still argues against indiscriminate biopsy, but this conclusion rests on hypothesis-generating data.

A selective approach to sampling offers an alternative. Malignancy is strongly linked with chronic ulcers, with mass-like lesions, with advanced age and with sites away from the prepylorus [8,22], and biopsy may accordingly be confined to such high-risk cases. For practical purposes, an ulcer may be regarded as suspicious when it shows one or more objective features: a diameter greater than 2–3 cm; heaped-up, rolled, or irregular margins; a firm or mass-like base; surrounding mucosal nodularity that does not resemble a simple acute peptic ulcer; a non-prepyloric (particularly body or cardia) location; or occurrence in an older patient with a chronic ulcer history. Making these criteria explicit is intended to render the selective-biopsy threshold reproducible rather than subjective. Importantly, perforated gastric ulcers should not be regarded as a single clinical entity: small, acute, benign-appearing ulcers differ fundamentally from large, chronic, or mass-like ulcers, the latter of which may in fact represent a perforated gastric carcinoma rather than a benign peptic ulcer. The current WSES guideline draws the same distinction, recommending resection with intraoperative frozen pathological examination for large gastric ulcers that raise suspicion of malignancy, provided the clinical state of the patient allows it [5]. Acute ulcers, particularly those of the pre-pyloric region, seldom conceal cancer and may not need to be sampled [6]. Frozen sections, moreover, are often unobtainable in the emergency setting and the tissue they provide may be of poor quality [15]. Even when the diagnosis is made, converting an operation for perforation into radical cancer surgery may not serve the patient well. Resection, anastomosis and lymphadenectomy can hardly be performed to the necessary standard in the presence of peritonitis. The safer course is to close the perforation and to arrange endoscopy once the patient has recovered. When malignancy is identified postoperatively, management should be individualized and multidisciplinary rather than defaulting to radical surgery after chemotherapy. Appropriate steps include staging with CT of the chest, abdomen, and pelvis; endoscopic reassessment with biopsy confirmation; and, when advanced disease is suspected, molecular characterization (HER2, MSI/MMR status, PD-L1 combined positive score, and, where relevant, CLDN18.2). Treatment is then determined at a multidisciplinary tumour board and may comprise perioperative chemotherapy followed by resection in fit patients with resectable disease, upfront surgery in selected cases, or palliative systemic therapy when the disease is metastatic or unresectable. The feasibility of this deferred pathway depends on adherence to postoperative endoscopy, which can be imperfect after emergency surgery; structured follow-up with scheduled surveillance endoscopy and clear discharge pathways is therefore essential so that deferring diagnosis does not translate into missed or delayed cancer detection. The clinical consequences of missing a gastric malignancy—stage progression, loss of a curative window, and worse survival—are serious, which is precisely why a selective strategy must be paired with reliable endoscopic surveillance rather than simple omission of biopsy. For these reasons, routine biopsy of every perforated gastric ulcer does not appear justified by the current evidence. Biopsy or an intraoperative frozen section examination is instead best reserved for the minority of patients with large, chronic, mass-like, or otherwise suspicious lesions, in whom the pre-test probability of malignancy is meaningfully higher and a positive result may influence the operative strategy.

Routine sampling adds cost and operating time and carries hazards of its own, above all for patients already critically ill with sepsis or peritonitis. Its value in the absence of clinical suspicion is thus open to question. Moreover, a histological confirmation of malignancy rarely alters the immediate surgical management, which typically involves simple closure or omental patching for perforation repair [23]. Judged by cost-effectiveness, routine biopsy offers little when the probability of cancer is so small. The point is reinforced by the fact that most gastric perforations arise from benign peptic ulcer disease, particularly where Helicobacter pylori infection and NSAID use are prevalent. Endoscopic surveillance after operation, once the patient has stabilized, provides a safer and more focused route to the detection and treatment of cancer in those who need it.

### Limitations

Several limitations qualify the strength of our conclusions. First, the evidence base is composed almost entirely of retrospective, single-arm, non-randomized studies, and only one study directly compared morbidity between biopsy and no-biopsy groups; the harm signal associated with biopsy therefore rests on limited comparative data. Second, the pooled malignancy proportion showed substantial heterogeneity (I^2^ = 72%), reflecting differences in era, geography, gastric cancer prevalence, and biopsy practice between studies, so the summary estimate should be interpreted with caution. Third, the search was restricted to two databases (PubMed/MEDLINE and Google Scholar) using title-anchored terms, which favours specificity over sensitivity and may have missed eligible studies indexed elsewhere; the absence of grey literature and non-English sources may further bias the malignancy estimate. Finally, and most importantly, the included studies did not consistently report ulcer size, chronicity, or macroscopic appearance, so perforated gastric ulcers had to be analyzed as a single entity. This precludes a formal subgroup analysis of the high-risk lesions in which a selective biopsy strategy is most likely to be worthwhile, and our recommendations should be read in that light. Several further limitations deserve emphasis. The complication comparison rested on markedly unequal groups: only 169 of 1122 patients did not undergo biopsy, and the single comparative study likewise had unequal arms, so the morbidity comparison is statistically fragile and vulnerable to confounding by indication. The restriction to PubMed/MEDLINE and Google Scholar with title-anchored terms—without Embase, Scopus, Web of Science, or the Cochrane Library—may have introduced selection bias by preferentially retrieving studies that carry these exact words in the title, and is more likely to have reduced sensitivity than earlier wording implied. The evidence base also included one non-peer-reviewed preprint and one postgraduate thesis; a sensitivity analysis excluding these sources left the pooled estimate within the same low range, but their inclusion is acknowledged as a limitation. Finally, study-level details such as the country attribution of individual reports and the derivation of the pooled demographics (with several studies not reporting sex and some means estimated from medians by the Hozo method) add further uncertainty to the summary figures.

## 5. Conclusions

On the evidence now available, sampling every perforated gastric ulcer at operation cannot be justified, the pooled rate of malignancy is low (3.1%) and morbidity may be increased. Where the ulcer is small and appears benign, closing the perforation and arranging endoscopic examination after recovery seems the safer policy. However, because the available evidence is retrospective, heterogeneous, and not stratified by ulcer characteristics, a selective biopsy or intraoperative frozen-section strategy remains reasonable for large, chronic, or suspicious gastric ulcers, in keeping with current WSES guidance. Adequately powered prospective studies that stratify by ulcer appearance are needed to define this subgroup more precisely. Because this recommendation rests primarily on retrospective, heterogeneous evidence and on a single comparative study of morbidity, it should be interpreted cautiously and regarded as support for a selective, individualized approach rather than a definitive rule against biopsy. If gastric cancer is identified on postoperative endoscopy, subsequent treatment should be individualized through multidisciplinary staging—rather than assuming radical surgery after chemotherapy in every case—and may involve perioperative chemotherapy followed by resection, upfront surgery, or palliative systemic therapy depending on stage and performance status.

## Figures and Tables

**Table 1:** Newcastle–Ottawa bias scores of the studies.

Author Name	Year	Study Design	Score (Out of 9)	Bias Risk
Amoli [6]	2024	Cross-sectional	7	Low
Bhaskar [7]	2019	Observational	6	Moderate
Janwanitchsthaporn [8]	2018	Retrospective	5	Moderate
Koca [9]	2024	Retrospective	6	Moderate
Leeman [10]	2013	Retrospective	6	Moderate
Madiba [11]	2005	Retrospective	5	Moderate
McGee [12]	1987	Retrospective	6	Moderate
Meenakshi [13]	2024	Prospective	5	Moderate
Oyomno [14]	2018	Retrospective	5	Moderate
Steyn [15]	2024	Prospective	7	Low
Trindade [16]	2025	Retrospective	6	Moderate
Turner [17]	1988	Retrospective	6	Moderate

**Table 2:** Clinical data of the selected studies.

Author	Year	Country	Patient No.	Female	Male	Mean Age	Biopsy+	Malign+	Malign%
McGee [12]	1987	US	91	NA	NA	57	66	6	9.1
Turner [17]	1988	US	107	NA	NA	49	63	1	1.6
Madiba [11]	2005	South Africa	72	10	62	43	72	2	2.8
Leeman [10]	2013	UK	44	NA	NA	57	33	3	9.1
Janwanitchsthaporn [8]	2018	Thailand	178	34	144	52	178	5	2.8
Oyomno [14]	2018	South Africa	93	NA	NA	39	67	2	3
Bhaskar [7]	2019	India	60	4	56	49	60	5	8.3
Amoli [6]	2024	Iran	175	75	100	70	175	0	0
Koca [9]	2024	Germany	135	56	79	55	110	0	0
Meenakshi [13]	2024	India	48	4	44	47	48	1	2.1
Steyn [15]	2024	South Africa	68	20	48	45	60	0	0
Trindade [16]	2025	Portugal	51	14	37	66	21	2	9.5

## Data Availability

All data generated or analyzed during this study are included in this published article and its Supplementary Information File.

## Supplementary Materials

The following supporting information can be downloaded at https://www.mdpi.com/article/10.3390/jcm15145460/s1, Table S1: PRISMA checklist.

## Abbreviations

CI	Confidence Interval
I^2^	I-squared Statistic
INPLASY	International Platform of Registered Systematic Review and Meta-analysis Protocols
NOS	Newcastle–Ottawa Scale
NSAID	Non-Steroidal Anti-Inflammatory Drug
PICOS	Population, Intervention, Comparator, Outcomes, Study Design
PRISMA	Preferred Reporting Items for Systematic Reviews and Meta-Analyses
RCT	Randomized Controlled Trial
WSES	World Society of Emergency Surgery
